# Domains of quality of life: results of a three-stage Delphi consensus procedure among patients, family of patients, clinicians, scientists and the general public

**DOI:** 10.1007/s11136-013-0578-3

**Published:** 2013-11-17

**Authors:** Suzanne Pietersma, Marieke de Vries, M. Elske van den Akker-van Marle

**Affiliations:** 1 Department of Medical Decision Making, Leiden University Medical Center, Post Zone J10-S, P.O. Box 9600, 2300 RC Leiden, The Netherlands; 2 Tilburg University, Tilburg, The Netherlands

**Keywords:** Quality of life, Delphi technique, Quality of health care, Cost–benefit analysis

## Abstract

**Purpose:**

Our key objective is to identify the core domains of health-related quality of life (QoL). Health-related QoL utility scales are commonly used in economic evaluations to assess the effectiveness of health-care interventions. However, health-care interventions are likely to affect QoL in a broader sense than is quantifiable with traditional scales. Therefore, measures need to go beyond these scales. Unfortunately, there is no consensus in the scientific literature on the essential domains of QoL.

**Methods:**

We conducted a three-stage online Delphi consensus procedure to identify the key domains of health-related QoL. Five stakeholder groups (i.e., patients, family of patients, clinicians, scientists and general public) were asked, on three consecutive occasions, what they perceive as the most important domains of health-related QoL. An analysis of existing (health-related) QoL and well-being measurements formed the basis of the Delphi-procedure.

**Results:**

In total, 42 domains of QoL were judged, covering physical, mental and social aspects. All participants rated ‘self-acceptance’, ‘self-esteem’ and ‘good social contacts’ as essential. Strikingly, mental and social domains are perceived as more essential than physical domains across stakeholders groups.

**Conclusions:**

In traditionally used health-related QoL utility measures, physical domains like ‘mobility’ are prominently present. The Delphi-procedure shows that health-related QoL (utility) scales need to put sufficient emphasis on mental and social domains to capture aspects of QoL that are essential to people.

## Introduction

Evaluating the benefits of health treatments can assist the allocation of scarce health-care resources by maximizing health benefits. Effectiveness of health-care interventions is currently preferably measured in terms of quality-adjusted life years [1, 2]. Quality-adjusted life years combine the quality and quantity of life into a one-dimensional outcome. Commonly used scales to assess health-related quality of life (QoL) are generic utility measures, like the EQ-5D [3]. These QoL measures provide utilities for different levels of a predefined set of domains (e.g., mobility). They focus on domains of QoL that can be expected to be affected by health-care interventions and are therefore often labeled as health-related QoL measures. An increasingly common critique is that such utility measures are too narrowly focused and do not capture all domains relevant to QoL [4, 5]. For example, these measures mainly focus on determining the physical effects of cure-related treatments and do not detect important effects of health-care interventions in the care-sector on mental and social domains of QoL [6, 7]. A worrying consequence is that the effects of health-care interventions are not as comprehensively captured as possible, which results in suboptimal measures of the effectiveness of health-care interventions. Therefore, measures need to go beyond these scales. Unfortunately, there is no consensus in the scientific literature on the core domains of QoL [5, 8–10]. To identify these core domains, we conducted a three-stage Delphi-procedure among different groups of experts. The current article outlines the outcomes of this Delphi-procedure.

### Delphi consensus procedure

Delphi consensus procedures have proven to be a valuable tool in gaining insight into health-related issues [11–13]. The selection of experts is critical to the success of the Delphi technique in providing in-depth understanding of scientific questions. Although a Delphi-procedure does not require representative sampling, it does require the cautious selection of panel members who are information- and experience-rich [14–16]. In many studies, multiple groups of experts are included to capture a broad spectrum of insights and information [13, 17]. Accordingly, we included five groups of experts: patients, family of patients, clinicians, scientists and the general public. We did not include two other groups that could be seen as informative, namely: board members of health-care insurers and policy makers, because workers in these professions are not expected to base their judgments on their own opinion but on existing information (e.g., scientific outcomes, statements of medical professionals). We will focus on the differences and similarities between all five groups.

## Methods

Before conducting a three-stage Delphi-procedure, we performed an extensive analysis of existing (health-related) QoL measurements to identify potential health-related QoL domains. This search provides solid input for the Delphi-procedure [18]. Our search was intended to be an open process that is able to identify all potential domains of health-related QoL (e.g., irrespective of level of abstractness). We did use a broad and general conceptual framework to structure the extensive number of domains we found during our search; we used the definition of health of the World Health Organization (WHO) [19] as guidance. That is, in accordance with the WHO definition, we perceive health-related QoL as a state of complete positive physical, mental, and social well-being. We used this broad conceptual framework, instead of a more specific theoretical model, because there is a degree of consensus within the scientific field of population health around this definition; concerning more specific theoretical models, there is much more debate and ambiguity [20]. We also used the criterion that domains could be influenced by health-care interventions (e.g., medical interventions, psychological interventions). The domains that we identified during our search formed the input for the first round of the Delphi-procedure.

Just as important, during the first questionnaire round of the Delphi-procedure, participants were encouraged to freely express their own beliefs. That is, they were stimulated to mention additional health-related QoL domains not found during the search or comment on the description of domains. These suggestions made by the participants during the first round were used to adjust the second-round questionnaire. Finally, the experts re-evaluated the second-round outcomes in the third round. This iterative approach allows participants to adjust their opinions when needed and to obtain feedback about the opinions of other people. All experts remained anonymous to minimize group pressure for conformity biases [21, 22].

### Analysis of existing measurements

We identified QoL domains that are part of existing health-related QoL utility measures and domains that are part of overall QoL, satisfaction or well-being measures. Health-related QoL utility measures mostly originate from an economical scientific tradition [23]. Overall QoL measures can originate from multiple backgrounds (e.g., medicine, psychology, sociology, economics).

#### (Health-related) QoL utility measures

The most commonly used health-related QoL utility measures, as previously identified [3, 24–26], are the EQ-5D, SF-6D, HUI2, HUI3, 15D, QWB-SA and AQoL. We examined these questionnaires and specified which domains are present in these measures.

#### General QoL measures

To get a broader and more diverse overview of domains of health-related QoL, we also looked at the QoL questionnaires included in the online archive of the Australian Centre on Quality of Life [27]. This database includes hundreds of questionnaires on health-related QoL, QoL, well-being, wellness and life satisfaction. Nine categories are specified (e.g., normal population, cognitive disability, children/adolescents). We selected the questionnaires from the category ‘normal population’ (762 questionnaires). For each scale, a short description and a reference to the original article is given in the online archive. We excluded questionnaires that still explicitly focused on specific groups (e.g., specific patient groups) and health-related utility measures already identified in the above-mentioned search. We divided all remaining questionnaires in two categories: (a) questionnaires that considered QoL in general and (b) questionnaires that considered specific domains within QoL (e.g., anxiety).

Based on the title of the questionnaire and a short description given in the online archive, we were able to see whether a scale considered QoL in general or specific domains. If a scale focuses upon QoL in general, we looked-up the original article to determine which domains were central in these measures.^1^ We identified 53 general questionnaires on QoL (of the 762 questionnaires in the online archive) that matched our criteria. For these questionnaires, we scrutinized the original article for the QoL domains. Concerning questionnaires that focus on specific QoL domains, we identified a total of 484 questionnaires that consider one or two specific domains of QoL (of the 762 questionnaires of the online archive). We used the title of the scales and general description to identify the specific QoL domains. We checked whether the scales were intended to be used in the general population.

#### Summarizing outcomes of analysis of existing measurements

An extensive list of domains was obtained. We excluded domains that were mentioned multiple times or domains that overlapped. Next, we excluded domains if they were so specific that they were only applicable to specific subgroups (e.g., satisfaction with meditation options). As mentioned above, the domains should also be expected to be influenced by health-care interventions. Finally, we ended up with 40 health-related QoL domains (see Table 1). These domains formed the input for the first round of the Delphi-procedure.

**Table 1:** Final list of domains based on analysis of existing (health-related) QoL measurements

	Domain	Definition of domain
1	No problems with performing activities of daily living	No problems with performing activities of daily living means that people are not limited in performing daily activities, such as work, study, housework, physical care, visiting family and/or leisure activities
2	Mobility	Mobility refers to how well one is able to get around indoors and outdoors. Mobility refers to walking, but also to one’s ability to use transportation options
3	Vitality	Vitality refers to one’s daily energy level. Potential fatigue and sleeping problems are also part of vitality
4	No somatic complaints	Somatic complaints refer to physical symptoms, discomfort, pain and other physically distressing issues that one can have
5	No problems related to communication	Problems related to communication refer to problems related to seeing, hearing and speaking
6	Experiencing positive emotions	Positive emotions cover the total range of possible positive emotions and feelings (for example, grateful, contented and happy)
7	Not experiencing negative emotions	Negative emotions cover the total range of possible negative emotions and feelings (for example, annoyed, nervous and restless)
8	Emotional control	Emotional control is the ability of people to recognize their own emotions and act on them when they deem it appropriate, not randomly and uncontrollably. It does not mean dismissing, blocking or ignoring one’s emotions
9	Emotional expressivity	Emotional expressivity is the tendency to show one’s own emotions to other people. It means that people do not try to cover up their emotions
10	Experience no depressed feelings	Depressed feelings refer to negative feelings such as feeling downhearted, sad and blue
11	Experience no anxious feelings	Anxious feelings refer to negative feelings such as feeling frightened, distressed, worried and uneasy
12	Mental capacity	Mental capacity refers to one’s potential to learn things. It involves things like thinking, memory and concentration
13	Control over unpleasant thoughts	Control over unpleasant thoughts is the ability to manage unpleasant thoughts by, for example, eliminating or blocking these thoughts
14	Mental balance	Mental balance is one’s calmness of mind. It is a relaxed outlook on life
15	Counterfactual thinking	Counterfactual thinking is the tendency to imagine alternatives to reality, that never actually happened. For example, “if only…” and “what if?” thoughts
16	Realistic beliefs	Realistic beliefs refer to: (a) the understanding that perfection is an impossible goal; (b) the ability to perceive reality accurately; (c) the ability to separate logical and rational from distorted and irrational; (d) the ability to avoid unrealistic expectations or wishful thinking
17	Optimism	Optimism is one’s tendency to expect and strive towards a positive outcome in life situations. It reflects the characteristics of hope and positive expectations
18	Self-acceptance	Self-acceptance is the complete acceptance of oneself as a valuable and pleasant human being—whether or not one is self-efficacious and whether or not others approve of or love you
19	Self-esteem	Self-esteem is one’s overall evaluation or appraisal of one’s own worth. It refers to the extent to which people like themselves in light of their assets and limitations, successes and failures, and their ability to cope with problems
20	Social contribution	Social contribution is helping, encouraging, and promoting the welfare of others. This may be done on your own or as a member of an organization such as a club, volunteer group or church
21	Social acceptance	Social acceptance is being accepted and feeling part of a community (like a social group, your neighborhood, your city)
22	Social support	Social support is the physical and emotional comfort given to or received from family, friends, co-workers and others
23	Personal relationships	Personal relationships refer to having positive relations/contacts with other people. It is the opposite of loneliness
24	Social intimacy	Social intimacy refers to the closeness of the relationships people have (for example, the relationship with one’s partner, children and/or friends)
25	Social functioning	Social functioning refers to one’s interpersonal functioning and social skills (for example, being able to respond and relate well to family, friends and/or groups). Good social skills are an important aspect of social functioning
26	Autonomy	Autonomy is the freedom people have to regulate their own life. Autonomous behaviors are those that are experienced as volitional and self endorsed. It means that one can make decisions without being coerced or pressured
27	Feeling in control	Feeling in control is the global feeling of having control over the important things in one’s own life. It is the feeling that you can determine yourself how to live your life (and that it is not determined by faith, coincidence, luck, your environment or other people). It is the opposite of feeling helpless
28	Environmental mastery	Environmental mastery is the ability to effectively manage one’s surrounding world and one’s life
29	Stress management	Stress management is the ability to effectively cope with one’s own stress level, or the ability to reduce one’s stress level or to prevent stress altogether
30	Willpower	Willpower is the ability of people to effectively regulate their own emotions, behavior and wants in order to reach long-term goals. It is the mental focus of people to actively pursue future ideals and not get distracted
31	Goal pursuit	Goal pursuit refers to one’s perseverance and motivation to achieve specific personally relevant goals.
32	Personal growth	Personal growth is one’s ability to make full use of one’s talents, capacities and potential
33	Personal achievement	Personal achievement refers to all that one has accomplished in life. It refers to all people’s successes and all goals that have been attained
34	Feeling competent and capable	Feeling competent and capable is the general feeling that one has the ability (knowledge, skills) to perform certain tasks or jobs adequately
35	Purpose in life	Purpose in life refers of having life goals and having a sense of direction in life. Those with high sense of purpose in life see their lives as meaningful
36	Being interested in one’s activities	Being interested in one’s activities refers to one’s involvement in one’s activities of daily life
37	Creativity	Creativity involves being original, imaginative, and inventive in one’s approach of all kinds of situations and activities
38	Satisfaction with living conditions (for example, financial situation)	Satisfaction with living conditions refers to one’s level of contentment with one’s income, things one owns such as a car or furniture, housing and/or one’s financial security
39	Satisfaction with daily activities (for example, work, hobbies, leisure time)	Satisfaction with daily activities refers to one’s level of contentment with one’s hobbies, recreational activities, ability of having free time, occupation, school activities and/or homemaking duties
40	Satisfaction with life roles	Satisfaction with life roles refers to one’s level of contentment with the way one can perform all relevant life roles, like the role of partner, mother/father, daughter/son, employee, friend etc.

### First round

#### First-round procedure and participants

Five different groups of participants were recruited to take part in the Delphi-procedure (see Table 2 for demographics): (a) *patients*—people who, at the moment of recruitment or in the previous year, had an acute/chronic physical/mental disease, were terminally ill or underwent fertility treatment; (b) *family members of patients*—people who had a family member who can be categorized as being a patient; (c) c*linicians*—people who had been working with patients/clients for at least 2 years. This was a diverse group; in the first round, the group consisted of 19 clinicians, 5 physiotherapists, 5 nurses and 5 psychologists. They had an average of 17.79 years of professional experience with patients/clients (SD = 10.41); (d) *scientific experts*—prominent researchers from all over the world on the topics QoL, well-being and health-related QoL. We personally approached the following people: (1) all first authors of the articles that are part of our analysis of existing general QoL measurements who stated in their online CV that QoL/well-being is their current main interest; (2) the editorial boards of five prominent scientific journals on QoL/well-being (i.e., Applied Psychology: Health and Well-Being, International Journal of Wellbeing, Psychology of Well-Being, Quality of Life Research, and Journal of Happiness studies). It is a mixed group; in the first round, the group consisted of 8 people with an economics background; 8 with a psychological background and the 16 remaining participants had varied backgrounds such as epidemiology, philosophy, psychiatry, marketing, public health; e) *general population*—people that did not fit the criteria of the above-mentioned groups.

**Table 2:** Respondents of three-stage Delphi-procedure

Participants	*N*	Age	Gender
Patients	38 [1] 37 (97 %) [2] 34 (92 %) [3]	*M* = 45.18; SD = 10.61 [1]	31 women; 7 men [1]
Family of patients	33 [1] 30 (91 %) [2] 28 (93 %) [3]	*M* = 48.85; SD = 14.68 [1]	23 women; 10 men [1]
Clinicians	34 [1] 31 (91 %) [2] 30 (97 %) [3]	*M* = 42.65; SD = 10.45 [1]	24 women; 10 men [1]
Scientist	32 [1] 32 (100 %) [2] 29 (91 %) [3]	*M* = 47.00; SD = 10.59 [1]	16 women; 16 men [1]
General population	33 [1] 36 (97 %) [2] 32 (97 %) [3]	*M* = 47.79; SD = 11.40 [1]	21 women; 12 men [1]

[1] round 1; [2] round 2; [3] round 3

Patients, family of patients and the general population were recruited by means of calls on Dutch websites of patient organizations and calls in local Dutch newspapers. Clinicians were personally approached. In exchange for participation in the total Delphi-procedure, we contributed 7.50 euro to a charity fund of their choice (except for scientific experts). All participants received a link to the first online Delphi questionnaire by e-mail.

#### First-round questionnaire

The first-round Internet-based questionnaire started with some background information about our project and about QoL. It was communicated that we defined health-related QoL as a state of complete positive physical, mental and social well-being (see definition of health of the World Health Organization [19]). We also emphasized that the domains should be able to be influenced by health-care interventions. Next, participants answered 40 questions; these covered the domains of health-related QoL that were identified during our analysis of existing measurements (see Table 1). Participants were asked to indicate for each domain to what extent they perceive each domain as an important part of health-related QoL (endpoints 1 [*not important*] to 4 [*very important*]). These 40 domains were categorized, based on content in five categories: physical, mental, social, domains on the interface of mental and social well-being, and remaining domains. These five categories were presented to participants in random order, and within each category the domains were also presented in random order. At the end of each list of domains belonging to a certain category, participants were encouraged to write down any comments. In addition, at the end of the questionnaire, participants were asked if they thought that domains were missing and if so, they were encouraged to write down these domain(s) and provide a short explanation. Finally, demographic questions were posed.

#### First-round analyses

First, we analyzed the answers given by the five groups on the 40 domains of health-related QoL. For all domains within each group, we determined whether or not there was consensus on the importance of a specific domain. In all the rounds, we used median scores (Mdn) to determine consensus. We did not use means, because means are more sensitive to extreme scores than Mdn scores, and are therefore less appropriate to determine the presence of consensus within groups [16]. We classified three outcomes in the first round: (a) consensus that a domain is highly important (Mdn = 4); (b) consensus that a domain is less or moderately important (Mdn < 3); (c) no consensus that a domain is highly important (Mdn ≥ 3 and <4). In addition, we analyzed the answers to all open-ended questions for all five groups together. Three different researchers looked at the open-ended answers to exclude interpretation bias [12, 13]. Suggestions that were mentioned by at least two participants were processed. This resulted in several changes (see Table 3).

**Table 3:** New, deleted, merged, and altered domains in the second and third round of Delphi-procedure

7 new domains	Definition of new domain
Independence	Independence means that people are not dependent on other people for their daily activities. It is the opposite of helplessness
Acceptation of the situation	Acceptation of the situation means that people accept their life as it is. They are flexible in dealing with changes in their lives
Enjoying the little things in life	Enjoying the little things in life means that people are able to be satisfied with little things in life and be satisfied with what they have
Being understood by one’s environment	Being understood by one’s environment means that people feel that they are understood and heard by those around them (for example family, friends, colleagues, social workers)
Meaningfulness	Meaningfulness is about concepts like spirituality, faith and religion
Future perspective	Future perspective revolves around people’s future expectations and prospects. These expectations can be positive and negative, but also uncertain or unknown
Satisfaction with the balance between obligations (e.g., work) and leisure (e.g., home situation)	Satisfaction with the balance between obligations and leisure refers to one’s level of contentment with how one can divide time between tasks. An important example is the balance between work and life

2 deleted domains	
Counterfactual thinking	
Environmental mastery	

5 merged domains into 2 new domains	Definition of merged domain
Social support Personal relationships Social intimacy	*Good social contacts* refer to having positive relations/contacts with and getting support from family, friends, colleagues and others. This can be physical support but also emotional support. It does not concern the number of contacts people have; it is about the fact that people feel supported. Having good social contacts means that people are not lonely
Willpower Goal pursuit	*Perseverance* is the tenacity with which people try to achieve important goals and the willpower to get you through difficult times

Alterations in wording	
Round 1	Round 2 and round 3
No problems with performing activities of daily living	*Being able to perform activities of daily living that are important to you* means that one is capable to perform daily activities, such as work, study, housework, physical care, visiting family and/or leisure activities
No somatic complaints	Somatic complaints refer to physical symptoms, discomforts, pain and other physically distressing issues that one can have. *Effectively dealing with these* *complaints* means that people experience little/no burden due to these complaints and that they are not restricted in their lives due to the complaints
No problems related to communication	Problems related to communication refer to problems related to seeing, hearing and speaking. *Effectively dealing with these problems means* that people experience little/no burden due to these problems and that they are not restricted in their lives due to the problems
Not experiencing negative emotions	Negative emotions cover the total range of possible negative emotions and feelings (for example, annoyed, nervous and restless). *Being well able to handle these negative emotions* means that people do not let these negative feelings control their lives but that these feelings are manageable
Experience no depressed feelings	Depressed feelings refer to negative feelings such as feeling downhearted, sad and blue. *Being well able to handle these depressed feelings* means that people do not let these depressed feelings control their lives but that these feelings are manageable
Experience no anxious feelings	Anxious feelings refer to negative feelings such as feeling frightened, distressed, worried and uneasy. *Being well able to handle these anxious feelings* means that people do not let these anxious feelings control their lives but that these feelings are manageable
Self-acceptance	Self-acceptance is the *complete acceptance of oneself the way you are. It revolves around the acceptance of your weaknesses and shortcomings*
Self-esteem	Self-esteem is one’s overall feeling of one’s own worth. *It refers to the extent to which people positively value themselves as a person*
Social contribution	*Meaningful contribution* means that people feel that they meaningfully contribute to their social environment or to society. This can involve (volunteer) work or involve helping others
Social acceptance	*Social acceptation by your environment* means that people have the feeling that they are being accepted by their environment and have the feeling that they are part of a community (like a social group, your neighborhood, your city)
Social functioning	*Social skills* refer to one’s interpersonal functioning (for example, being able to respond and relate well to family, friends and/or groups)

In round 2 and 3, the total number of included domains is maximally 42

The italic parts are the alterations in wording in round 2 and 3 in comparison with round 1

### Second round

#### Second-round procedure and participants

All people who participated in the first round were invited to participate in the second round. This round started 6 weeks after the first. Each group was asked to re-rate the domains of the first round, for which a consensus on whether they were highly important or not was not reached in that group (Mdn ≥ 3 and <4), and to rate the added and altered domains. For domains that were re-rated, a summary report was presented to the participant in which their answers to the first round, the average of the group and a frequency chart of the group’s answers were shown.

#### Second-round questionnaire

In the second-round questionnaire, the domains were also ordered in five categories. In the second-round questionnaire, we used a 7-point scale (endpoints 1 [*totally not important*] to 7 [*very important*]). We included a more detailed response scale to get more variance in the answers.

#### Second-round analyses

In this round, we determined consensus by means of both Mdn scores and Inter-Quartile Deviations (IQDs). IQDs are commonly used to determine consensus [11, 16]. We included IQDs because our 7-point scale allowed for a meaningful interpretation of this outcome. IQD represents the distance between the twenty-fifth percentile and the seventy-fifth percentile values in opinions, with a smaller IQD indicating larger consensus. An IQD ≤ 1 can be considered as good consensus on a 7-point Likert scale [15]. We classified three outcomes: (a) consensus and agreement that a domain is highly important (IQD ≤ 1 and Mdn ≥ 6); (b) consensus that a domain is less to moderately important (IQD ≤ 1 and Mdn ≤ 5); (c) no consensus (IQD > 1).

### Third round

#### Third-round procedure and participants

All people who participated in round two were invited to participate in round three. This round started 4 weeks after the previous round. Each group was asked to re-rate the domains of round two for which no consensus was reached. Again, a summary report of the results of the previous round was presented.

#### Third-round questionnaire

No domains were changed or added to the third-round questionnaire compared with the second-round questionnaire. The response scales were identical to round two. In the third round, we also asked respondents to indicate which five domains they perceived as most important aspects of QoL of all 42 domains. Next, participants were asked to rank these five domains from least important to most important.

#### Third-round analyses

The same criteria as used in the second round (i.e., Mdn scores and IQDs) were applied to determine consensus and agreement. In addition, for each group, we determined which domains were mentioned most often in the list of five most important domains.^2^

## Results

We analyzed the results for the five groups separately. In Table 4, the results of all three Delphi-procedure rounds are presented.

**Table 4:** Means and other results of Three-Stage Delphi-procedure for all five groups

		Patients	Family of patients	Clinicians	Scientists	General population
*Physical domains*						
1	Being able to perform activities of daily living that are important to you	**6.51 [2]**	6.15 [1]	**5.94 [2]**	**6.03 [2]**	**6.24 [2]**
2	Mobility	**6.31 [1]**	6.15 [1]	5.61 [2]	*5.20 [2]*	5.88 [2]
3	Vitality	6.05 [2]	**6.42 [1]**	5.80 [3]	**5.72 [3]**	5.91 [–]
4	Effectively dealing with somatic complaints	6.14 [2]	5.79 [3]	5.61 [2]	5.14 [–]	5.70 [2]
5	Effectively deal with problems related to communication	5.82 [–]	*5.46 [3]*	5.65 [2]	5.34 [3]	5.73 [2]
6	Independence	**6.59 [2]**	**6.27 [2]**	5.66 [3]	4.97 [–]	**6.06 [3]**
*Mental domains*						
7	Positive emotions	**6.22 [1]**	6.13 [2]	5.83 [–]	**6.18 [1]**	5.91 [3]
8	Being well able to handle negative emotions	6.03 [–]	5.70 [2]	5.77 [2]	5.48 [–]	5.73 [2]
9	Emotional control	5.73 [2]	5.63 [2]	*5.20 [3]*	*5.25 [2]*	*5.30 [2]*
10	Emotional expressivity	5.59 [2]	5.47 [2]	*5.10 [2]*	*4.38 [1]*	4.91 [–]
11	Being well able to handle depressed feelings	5.89 [2]	6.04 [3]	5.61 [2]	5.38 [–]	5.91 [2]
12	Being well able to handle anxious feelings	5.94 [–]	6.11 [–]	5.39 [2]	*5.24 [3]*	5.76 [2]
13	Mental capacity	5.99 [1]	6.04 [3]	*5.30 [3]*	*4.69 [3]*	*5.18 [2]*
14	Control over unpleasant thoughts	5.91 [–]	5.75 [3]	5.52 [2]	5.03 [–]	5.58 [2]
15	Mental balance	**6.22 [1]**	**6.20 [1]**	5.81 [2]	5.41 [–]	**6.00 [2]**
16	Realistic beliefs	6.03 [2]	5.71 [3]	*5.20 [3]*	4.69 [–]	*5.30 [2]*
17	Optimism	**6.31 [1]**	6.13 [2]	5.63 [3]	*5.31 [3]*	5.94 [–]
18	Self-acceptance	**6.45 [1]**	**6.42 [1]**	**6.38 [1]**	**5.66 [2]**	**6.47 [1]**
19	Self-esteem	**6.36 [1]**	**6.36 [1]**	**6.33 [1]**	5.69 [–]	**6.31 [1]**
20	Acceptation of the situation	**6.38 [2]**	**6.36 [3]**	5.33 [–]	5.10 [–]	**5.97 [3]**
21	Enjoying the little things in life	**6.65 [2]**	**6.33 [2]**	5.90 [2]	**5.62 [3]**	**6.36 [2]**
*Social domains*						
22	Meaningful contribution	5.78 [2]	5.70 [2]	5.87 [–]	*5.22 [2]*	5.79 [2]
23	Social acceptation by your environment (e.g., people from your neighborhood)	5.62 [–]	5.68 [3]	5.61 [2]	5.07 [–]	*5.25 [3]*
24	Social skills	5.81 [2]	5.71 [3]	5.71 [2]	5.10 [–]	5.72 [–]
25	Good social contacts	**6.24 [2]**	6.20 [2]	**6.17 [3]**	5.93 [–]	5.91 [2]
26	Being understood by one’s environment	6.12 [–]	5.89 [–]	5.55 [2]	4.72 [–]	5.52 [2]
*Interface of mental and social domains*						
27	Autonomy	**6.22 [1]**	**6.26 [1]**	**6.33 [1]**	5.62 [–]	**6.26 [1]**
28	Feeling in control	6.13 [1]	6.07 [3]	5.65 [2]	5.41 [3]	5.61 [2]
29	Stress management	5.81 [2]	5.60 [2]	5.45 [2]	5.21 [–]	5.60 [–]
30	Personal growth	5.53 [1]	5.50 [3]	5.71 [2]	5.62 [–]	5.42 [2]
31	Personal achievement	5.35 [2]	*5.40 [2]*	*5.16 [2]*	5.07 [–]	*4.94 [2]*
32	Feeling competent and capable	6.11 [2]	6.07 [3]	**6.03 [3]**	**5.63 [2]**	5.79 [2]
33	Purpose in life	6.03 [1]	5.89 [1]	**6.06 [2]**	**6.18 [1]**	**6.31 [1]**
34	Being interested in one’s activities	5.95 [2]	5.70 [2]	5.84 [2]	**5.91 [1]**	5.91 [3]
35	Creativity	5.35 [2]	5.40 [2]	4.97 [–]	*4.52 [3]*	*5.03 [3]*
36	Perseverance	6.21 [2]	5.93 [–]	5.58 [2]	4.79 [–]	5.58 [2]
37	Meaningfulness	5.43 [2]	4.93 [–]	5.70 [–]	5.31 [–]	5.53 [–]
38	Future perspective	6.05 [2]	5.96 [3]	5.90 [2]	4.79 [–]	*5.36 [2]*
*Other domains*						
39	Satisfaction with living conditions (e.g., financial situation)	5.76 [3]	5.86 [3]	5.68 [2]	*5.19 [2]*	5.42 [2]
40	Satisfaction with daily activities (e.g., work, hobbies, leisure time)	6.16 [2]	**6.36 [1]**	**6.07 [3]**	**5.66 [2]**	**6.05 [1]**
41	Satisfaction with life roles	**6.22 [1]**	6.10 [1]	**6.18 [1]**	**6.13 [1]**	5.69 [3]
42	Satisfaction with the balance between obligations (e.g., work) and leisure (e.g., home situation)	6.22 [2]	**6.25 [3]**	**6.13 [2]**	*5.14 [3]*	5.72 [–]

[1] consensus is achieved in round 1; [2] consensus is achieved in round 2; [3] consensus is achieved in round 3; [–] no consensus is reached

Italics values: consensus is reached that a domain is not important; values without emphasis: consensus is reached that domain is an important aspect of QoL

Bold values are the top 10 domains (highest means) for each group; we only considered domains for which consensus was reached that domain is important aspect of QoL. For the group patients, we marked 13 domains because multiple domains had the same mean

In round 1, a response scale with endpoints 1 and 4 was used; in rounds 2 and 3, a response scale with endpoints 1 and 7 was used. To make the results of all three rounds comparable, we have multiplied the results of round 1 with 7/4th

### First-round results

Patients and family members of patients already agreed in the first round on the high importance of a quarter of the presented domains. The other groups reached consensus on the high importance of only a few domains in round one. There is no domain for which all five groups reached consensus on the high importance. However, all groups, except the scientists, agreed on the high level of importance of the domains: self-acceptance, self-esteem and autonomy.

### Second-round results

The outcomes show that especially the scientists perceive much fewer domains as highly important, than the other groups. In addition, both scientists and the general population reached consensuses that several domains are not important, while the other groups mainly reported reaching consensus on the high level of importance of domains. Looking at specific domains, differences between groups are also clearly present. For example, the domain ‘emotional control’ is perceived as highly important by patients and family of patients, but as not highly important by scientists and the general population. There are also similarities between the groups. For example, almost all groups agree on the high level of importance of ‘being able to perform activities of daily living that are important to you’ and ‘enjoying the little things in life’.

### Third-round results

#### Third-round results of five groups

A striking finding is the extensive absolute number of domains for which no consensus is attained by scientific experts. They did not reach consensus on 20 domains, while for the other groups, this was the case for less than 10 domains. The third round mainly proved effective in reaching consensus on a large absolute number of domains for family of patients.

At the end of round three, we wanted to get a better understanding of the similarities and differences between all five groups. Because many groups perceived a large number of domains as highly important, we made a further selection—we looked at the 10 domains with the highest mean ratings on which consensus and agreement was attained that they were highly important (within each group).^3^ This provided a rather diverse picture. The five groups strongly agree on ‘self-acceptance,’ as this domain is rated highly by all groups. The next domains are rated highly by the majority of groups (i.e., three or four groups): being able to perform activities of daily living that are important to you, independence, mental balance, self-esteem, acceptation of the situation, enjoying the little things in life, autonomy, purpose in life, satisfaction with daily activities and satisfaction with life roles.

#### Five most important domains

In Table 5, the 10 domains that were most frequently mentioned in the list of five most important domains of each group are given. ‘Self-esteem’ and ‘good social contact’ were part of the list of all groups. The following domains were part of the list of the majority of the groups (i.e., three or four groups): self-acceptance, independence, being able to perform activities of daily living that are important to you, optimism, autonomy and purpose in life.^4^ All these outcomes closely resemble the described outcomes based upon the highest mean ratings.

**Table 5:** Frequencies of domains mentioned in the list of five most important domains in the third round of the Delphi-procedure

	Patients	Family of patients	Clinicians	Scientists	General population
*In top 10 of all groups*					
Self-esteem	6	6	6	6	11
Good social contacts	7	10	8	7	7
*In top 10 of four groups*					
Self-acceptance	7		12	6	15
Independence	15	11	6		7
*In top 10 of three groups*					
Being able to perform activities of daily living that are important to you	15	6	6		
Optimism		6		6	7
Autonomy	8		6	6	
Purpose in life		6		12	6
*In top 10 of two groups*					
Mental balance	8	6			
Acceptation of the situation		11			6
Enjoying the little things in life			6		10
Meaningfulness			9		8
Satisfaction with daily activities (e.g., hobbies, leisure time)	6			6	
Satisfaction with the balance between obligations (e.g., work) and leisure (e.g., home situation)		6	10		
*In top 10 of one group*					
Mobility	9				
Vitality			6		
Positive emotions				7	
Being well able to handle negative emotions				6	
Realistic beliefs	6				
Being understood by one’s environment	6				
Feeling in control	8				
Stress management			6		
Personal growth				8	
Satisfaction with living conditions (e.g., financial situation)	7				
Perseverance					6

The numbers indicate how frequently a domain is mentioned in the list of five most important domains of QoL. The frequencies are calculated for each group separately

We did not use an absolute top 10 of each group (range between top 9 and top 13). Because the frequencies are equal for a number of domains, we choose to use a cut-off point; all domains that are mentioned more than 5 times are listed. This results approximately in a top 10

## Discussion

The Delphi-procedure we performed aimed to identify the essential domains of QoL that are important in the context of health-care interventions. Consequently, our Delphi-procedure shows which domains should potentially be included in generic preference-based (utility) scales to comprehensively measure health-related QoL. Generic preference-based measures are used for evaluation of (cost-) effectiveness of health-care interventions, capturing meaningful within-person change over time when it occurs.

Five different groups of experts rated more than 40 potential domains of health-related QoL. The results showed that all five groups agreed on few domains. That is, only ‘*self*-*acceptance*’ is part of the highest mean ratings of all groups. When looking at the list of five most important domains, ‘*self*-*esteem*’ and ‘*good social contacts*’ are the only two domains on which all five groups agree that they are highly important. Interestingly, these domains cover aspects that can be classified as mental and social phenomena and not as typical physical domains that are part of health utility measures, like the EQ-5D and SF-6D (i.e., mobility, vitality, dealing with somatic complaints). Looking at the more extensive lists of domains that the majority of the groups see as highly important (i.e., three or four groups), we again see that most of the domains concern mental and social phenomena. Moreover, the typical physical domains used in health-related QoL utility measures (like ‘vitality’ and ‘mobility’) are not present in these more extensive lists. In sum, mental and social domains are perceived as more essential than physical domains across all five stakeholders groups. This conclusion can have practical implications for future (cost-) effectiveness studies concerning health-care interventions. It can be stated that adding (more) mental and social domains to existing health-related QoL utility measures will result in a more comprehensive operationalization of health-related QoL. This will ultimately facilitate the allocation of health-care resources to interventions that are most effective in increasing people’s (health-related) QoL in relation to their costs.

An important point of consideration is that we know little about what participants are thinking when they provide scores on health states in existing health-related QoL measures. It is possible that respondents when faced with a health state with substantial physical impairments (e.g., impaired vision, impaired cognition), they might readily infer that in such a state social interaction is limited and self-esteem is low and therefore provide a low score on that state. So it is possible that existing generic preference-based measures already implicitly, to some extent, include social and mental domains. Our Delphi-procedure does, however, show the importance of providing more explicit attention to social and mental domains.

### Limitations

We chose to present participants at the start of the Delphi study with a list of domains that is extracted from existing questionnaires. Participants did have the opportunity to add, delete or alter domains. However, by presenting participants with a predefined set of domains, we might have led the participants’ way of thinking about health-related QoL. An alternative would have been to simply ask participants to list essential domains of health-related QoL. We chose to use a predefined set of domains to give our study a solid base, [18] and we believe that providing no list of domains could have been too cognitively demanding, resulting in a small list of domains that only include the domains that come to mind most easily. Second potential point of concern is that we did not explicitly select a specific theoretical framework at the beginning of our Delphi-procedure to guide our study. We used a broad conceptual framework—the WHO definition of health. By using existing measures as input for our Delphi-procedure, we have implicitly incorporated the specific theoretical frameworks of health-related QoL underlying these existing measures. For undertaking future steps, it can be helpful to use a more specific theoretical model. That is, the next step is to narrow down the list of 25 domains (see Table 5), resulting from this Delphi study, to create a new and comprehensive measure of health-related QoL. Utility measures require a limited number of questions/domains to be included [28]. A more detailed theoretical model on health-related QoL could provide informative input for this search process.

### Theoretical issues

What sort of theoretical issues does a more specific theoretical model on health-related QoL need to resolve? An important theoretical issue is that the domains that are part of our Delphi-procedure differ in level of abstractness. Some concern people’s general life views (i.e., feeling an autonomous person) and others are very concrete and objectively measurable (i.e., being able to walk). It is possible that some specific domains are part of another larger and more abstract domain [29]. In addition, the domains also differ in the extent to which they are ‘proximal’ or ‘distal’. Proximal refers to domains such as activities of daily living, while distal refers to more fundamental domains that influence the extent to which one can perform activities of daily living. Functional limitations may for example influence being capable of engaging in self-care. A second theoretical issue concerns social domains; some scientists suggest that social interaction should be omitted from the domains included in a measure of (health-related) QoL. It is argued that social interaction affects health and health affects social interaction, and therefore, social interaction should be measured separately [30]. A final theoretical issue that needs to be addressed is whether our new utility scale is intended to capture people’s capabilities (e.g., coping abilities) versus their functioning. This distinction is made in the Capability Theory of Sen [31]. Focusing on capabilities is a paradigm that is given increasing attention in health economics [32]. We see strong added value for focusing on people’s capabilities; it makes it possible to capture the extent to which people are able to autonomously cope with life’s ever changing physical, mental and social challenges.

### Future steps

In order to develop a new QoL utility measure, several follow-up steps need to be taken to narrow down the number of domains. We believe that the first step should be to make a rough selection of health-related QoL domains (present in the Delphi-procedure) and construct concrete questions that capture these domains. The next step will be testing these questions in a large sample of respondents. For example, to test which domains, if any, overlap in a factor analysis and see how the domains correlate with existing QoL/well-being measures to determine which domains have the best level of validity in capturing physical, mental and social phenomena of QoL. We will include a diverse array of measures: questionnaires on health-related QoL and more general QoL/well-being measures and both multi-attribute and one-dimensional questionnaires. This enables us to create a smaller, more operationalizable, set of domains.

## Conclusion

Inevitably, health-care resources are scarce. Evaluating the benefits of health-care interventions assists the allocation of these scarce resources and helps to maximize health benefits. An increasingly common critique is that traditionally used scales are too narrowly focused, resulting in suboptimal measures of effectiveness in which not all relevant domains of QoL are captured. Therefore, measures need to go beyond these scales. Unfortunately, there is no consensus on the core domains of QoL. The current three-stage Delphi consensus study among patients, family of patients, clinicians, scientists and the general public shows that measures need to put more emphasis on mental and social domains to capture aspects of QoL that are essential to people.

## Footnotes

1 Before looked-up the original article, we applied additional selection criteria; we excluded scales if: (a) questionnaires did not include domains, but only overall QoL questions; (b) the reference article was not published in English; (c) no reference to a peer-reviewed article was present; (d) a more updated version of the scale was mentioned in the database; (e) questionnaires were published before 1990. The last criterion is applied because we wanted to exclude questionnaires that were developed in a time when the definition of (health-related) QoL was stricter. Although we can not objectively identify an absolute date, we believe that 1990 is a good cut-off point, because since this date the discussion of the definition of (health-related) QoL intensified [23].

2 We also analyzed how participants ranked the five most important domains. If they ranked a domain as number 1 it got the value 5, if it was ranked number 2 it got the value 4, if it was ranked 3 it got the value 3, if it was ranked 4 it got the value 2 and if it was ranked 5 it got the value 1 (i.e., the resulting scores are named weighted means). This way we interpret the distances between domain 1 and domain 2 to be equal to the distance between domain 2 and 3, and so on.

3 In Table 4 we also present the means for all domains to get additional input on the relative importance of all domains (e.g., what domain for which consensus is reached is seem as most important?). Mdn scores provide less differentiation [16].

4 The weighted means based on the participants’ ranking of the five most important domains resulted in a very similar list. It is just a bit more extensive; it includes also the domains: mental balance, feeling in control and enjoying the little things in life.
